# Postoperative complications of reverse total shoulder arthroplasty: a multicenter study in Japan

**DOI:** 10.1016/j.jseint.2023.04.002

**Published:** 2023-04-20

**Authors:** Kenta Inagaki, Nobuyasu Ochiai, Eiko Hashimoto, Fumiya Hattori, Yu Hiraoka, Shohei Ise, Yohei Shimada, Daisuke Kajiwara, Koji Akimoto, Yasuhito Sasaki, Yu Sasaki, Norimasa Takahashi, Koji Fujita, Seiji Ohtori

**Affiliations:** a Department of Orthopaedic Surgery, Chiba University Hospital, Chuou-ku, Chiba-city, Chiba, Japan; b Department of Orthopaedic Surgery, Seirei Sakura Citizen Hospital, Sakura-city, Chiba, Japan; c Department of Orthopaedic Surgery, Chiba Rosai Hospital, Ichihara-city, Chiba, Japan; d Department of Orthopaedic Surgery, Sanmu Medical Center, Sanmu-city, Chiba, Japan; e Funabashi Orthopedic Hospital, Funabashi-city, Chiba, Japan; f Department of Orthopaedic Surgery, Chiba Medical Center, Chuou-ku, Chiba-city, Chiba, Japan

**Keywords:** Reverse total shoulder arthroplasty, Complication, Scapular otching, Survival rate, Acromial fracture, Neurological disorder

## Abstract

**Background:**

Reverse total shoulder arthroplasty (RSA) has been approved since 2014 in Japan, and the number of RSA cases has been accumulating. However, only short-to medium-term outcomes have been reported, with a small number of case series, because of its short history in Japan. This study aimed to evaluate complications after RSA in hospitals affiliated with our institute, with comparison to those in other countries.

**Methods:**

A multicenter retrospective study was performed at 6 hospitals. In total, 615 shoulders (mean age: 75.7 ± 6.2 years; mean follow-up: 45.2 ± 19.6 months) with at least 24 months of follow-up were included in this study. The active range of motion was assessed pre-and postoperatively. The 5-year survival rate was evaluated for reoperation for any reason in 137 shoulders with at least 5 years of follow-up using Kaplan–Meier analysis. Postoperative complications were evaluated, including dislocation; prosthesis failure; deep infection; periprosthetic, acromial, scapular spine, and clavicle fractures; neurological disorders; and reoperation. Furthermore, imaging assessments, including scapular notching, prosthesis aseptic loosening, and heterotopic ossification were evaluated on postoperative radiography at the final follow-up.

**Results:**

All range of motion parameters were significantly improved postoperatively (*P* < .001). The 5-year survival rate was 93.4% (95% confidence interval: 87.8%-96.5%) for reoperation. Complications occurred in 256 shoulders (42.0%), with reoperation in 45 (7.3%), acromial fracture in 24 (3.9%), neurological disorders in 17 (2.8%), deep infection in 16 (2.6%), periprosthetic fracture in 11 (1.8%), dislocation in 9 (1.5%), prosthesis failure in 9 (1.5%), clavicle fracture in 4 (0.7%), and scapular spine fracture in 2 (0.3%). Regarding imaging assessments, scapular notching was observed in 145 shoulders (23.6%), heterotopic ossification in 80 (13.0%), and prosthesis loosening in 13 (2.1%).

**Conclusion:**

This is the first large case series to investigate the complications after RSA in Japan, and the overall frequency of complications after RSA was similar to that in other countries.

Since its first description by Grammont et al in 1987,^14^ reverse total shoulder arthroplasty (RSA) has become a common surgery for the restoration of shoulder function in patients with cuff tear arthropathy and irreparable rotator cuff tears, and its indication has been expanded because of its good clinical outcomes.^2^^,^^4^^,^^10^^,^^19^^,^^25^^,^^33^ RSA was developed to achieve 2 biomechanical concepts: lowering of the humerus and medialization of the center of rotation at the glenoid component. Its design has the advantage of tensioning the deltoid muscle to increase functional strength. RSA has been performed by qualified surgeons in Japan, under restricted guidelines to decrease perioperative complications, since it was approved by the Ministry of Health and Welfare in 2014. The annual number of RSA cases is increasing in Japan, from 630 shoulders in 2014 to 1725 shoulders in 2019.

However, higher complication rates have been reported with RSA than with anatomic total shoulder arthroplasty and there are unique complications with RSA, such as fatigue fractures of the acromion and scapular spine.^20^^,^^31^ Furthermore, the complication rate for RSA is higher in the early postoperative setting and equalizes in mid- and long-term settings.^3^^,^^10^ Although several articles have reported good long-term outcomes for RSA in other countries,^2^^,^^3^^,^^10^^,^^11^ there are only reports on short-to medium-term outcomes in a few small case series studies because of its short history in Japan.^21^ Accordingly, the frequency of postoperative complications in RSA in Japan remains unknown. As implants are often designed for Western patients despite population differences in body size and race, it is important to investigate the occurrence of complications after RSA in Japan.

Therefore, this study aimed to evaluate complications after RSA in hospitals affiliated with our institute, with comparison to those in other countries.

## Materials and methods

### Study population

This multicenter retrospective study was performed at 6 hospitals affiliated with Chiba University Hospital (Funabashi Orthopedic Hospital, Chiba Medical Center, Seirei Sakura Citizen Hospital, Sanmu Medical Center, and Chiba Rosai Hospital). We included patients who underwent RSA between April 2014 and December 2020 with at least 24 months of follow-up. All procedures were performed by trained shoulder surgeons with RSA qualifications in Japan. After approval from our institutional review board, informed consent was obtained by placing a public document on the homepage of each participating hospital. Patients who did not wish to participate in the study (indicated by opting-out) were excluded.

A total of 817 shoulders underwent RSA during the study period. None of the patients declined to participate in this study. We excluded 202 shoulders because of inadequate follow-up; the remaining 615 shoulders were enrolled in this study.

### Assessments

The active range of motion (ROM) was assessed pre-and postoperatively (at the final follow-up), as a clinical outcome. External rotation (ER) in the drooping arm position was defined as the first plane (1st ER), and ER and internal rotation (IR) at 90° of shoulder abduction as the second plane (2nd ER and IR, respectively). The ROM at the final follow-up and prosthesis type (in-lay/on-lay) were compared between patients with and without scapular notching, which is considered to be frequent and associated with poor clinical outcomes.^29^^,^^32^

Postoperative complications, such as dislocation; prosthesis failure; deep infection; periprosthetic, acromial, scapular spine, and clavicle fractures; neurological disorders; and reoperation, were evaluated. Additionally, among reoperations (for any reason), the number of cases requiring revision surgery (involving implant replacement) was examined. Deep infection was defined as infection requiring débridement. The indication for débridement was determined by each surgeon with consideration of the results of the physical examination, blood tests, imaging findings, and bacterial culture tests. Acromial, scapular spine, and clavicle fractures were diagnosed by radiography; computed tomography (CT) (Revolution Maxima; GE Healthcare, Tokyo, Japan) was added if pain in these areas appeared and a fracture was suspected. We excluded 3 shoulders with traumatic clavicle fractures.

Imaging assessments, including scapular notching (Sirveaux classification^30^), prosthesis aseptic loosening, and heterotopic ossification, were evaluated on postoperative radiography at the final follow-up. Prosthesis loosening was defined as a radiolucent line >2 mm. All imaging data were evaluated by 2 orthopedic surgeons at each hospital, and the consensus was used for analysis. Differences in opinion were resolved through discussion or by referring to a third person's opinion.

### Statistical analysis

The paired *t* test was used to compare pre- and postoperative ROM parameters. The independent *t* test was used to compare postoperative ROM parameters between patients with and without scapular notching. The Fisher’s exact test was used to examine the difference in the frequency of scapular notching between in-lay and on-lay types. Additionally, Kaplan–Meier analysis was used to estimate the 5-year survival rate for reoperation for any reason and revision surgery in 137 shoulders with at least 5 years of follow-up. All statistical analyses were performed using EZR software (Saitama Medical Center, Jichi Medical University, Saitama, Japan). *P* values < .05 were considered statistically significant.

## Results

In total, 615 shoulders (mean age: 75.7 ± 6.2 years, mean follow-up: 45.2 ± 19.6 months, male/female: 230/385 shoulders) were enrolled in this study. All of the patients completed clinical and radiological follow-ups. Patient demographics are shown in Table I. The most common primary disease for RSA was cuff tear arthropathy (196 shoulders, 31.9%), followed by irreparable rotator cuff tear (142 shoulders, 23.1%) and acute proximal humeral fractures (83 shoulders, 13.5%).

**Table I:** Patient demographics.

Age (mean ± SD, yr)	75.7 ± 6.2 (51-92)
Follow-up (mean ± SD, mo)	45.2 ± 19.6 (24-96)
Sex (male/female, shoulders)	230/385
Side (right/left, shoulders)	416/199
Primary disease (shoulders, %)	
CTA	196 (31.9%)
Irreparable RCT	142 (23.1%)
Acute proximal humeral fracture	83 (13.5%)
Fracture sequela	61 (9.9%)
Osteoarthritis	48 (7.8%)
Rotator cuff retear	38 (6.2%)
Rheumatoid arthritis	25 (4.1%)
Other	22 (3.6%)
Medical history (shoulders, %)	
Diabetes mellitus	90 (14.6%)
Parkinsonism	12 (2.0%)
Weight-bearing	2 (0.3%)

*SD*, standard deviation; *CTA*, cuff tear arthropathy; *RCT*, rotator cuff tear.

Surgical details are shown in Table II. The deltopectoral approach was used in almost all patients. Additionally, an in-lay type prosthesis was used in the majority of cases (386 shoulders, 62.8%), while a cemented stem was used in 226 (36.8%) shoulders. Combined procedures included subscapularis tendon repair (440 shoulders, 71.5%), the modified L’Episcopo procedure^5^^,^^17^ (46 shoulders, 7.8%), and latissimus dorsi transfer^13^ (12 shoulders, 2.0%). Bone grafts for the glenoid were used in 108 (17.6%) shoulders.

**Table II:** Prosthesis and surgical methods.

Deltopectoral approach (n, %)	610 (99.2%)
In-lay type (n, %)	386 (62.8%)
Aequalis	187 (30.4%)
TM reverse shoulder system	109 (17.7%)
Delta Xtend	80 (13.0%)
SMR	7 (1.1%)
Medacta	3 (0.5%)
On-lay type (n, %)	229 (37.2%)
Ascend flex	137 (22.3%)
Equinoxe	72 (11.7%)
Comprehensive	20 (3.3%)
Cemented stem (n, %)	226 (36.8%)
Subscapularis repair (n, %)	440 (71.5%)
Modified L’Episcopo (n, %)	46 (7.8%)
LD transfer (n, %)	12 (2.0%)
RSA with bone graft (n, %)	108 (17.6%)

*LD*, latissimus dorsi; *RSA*, reverse total shoulder arthroplasty.

**Figure 1:**
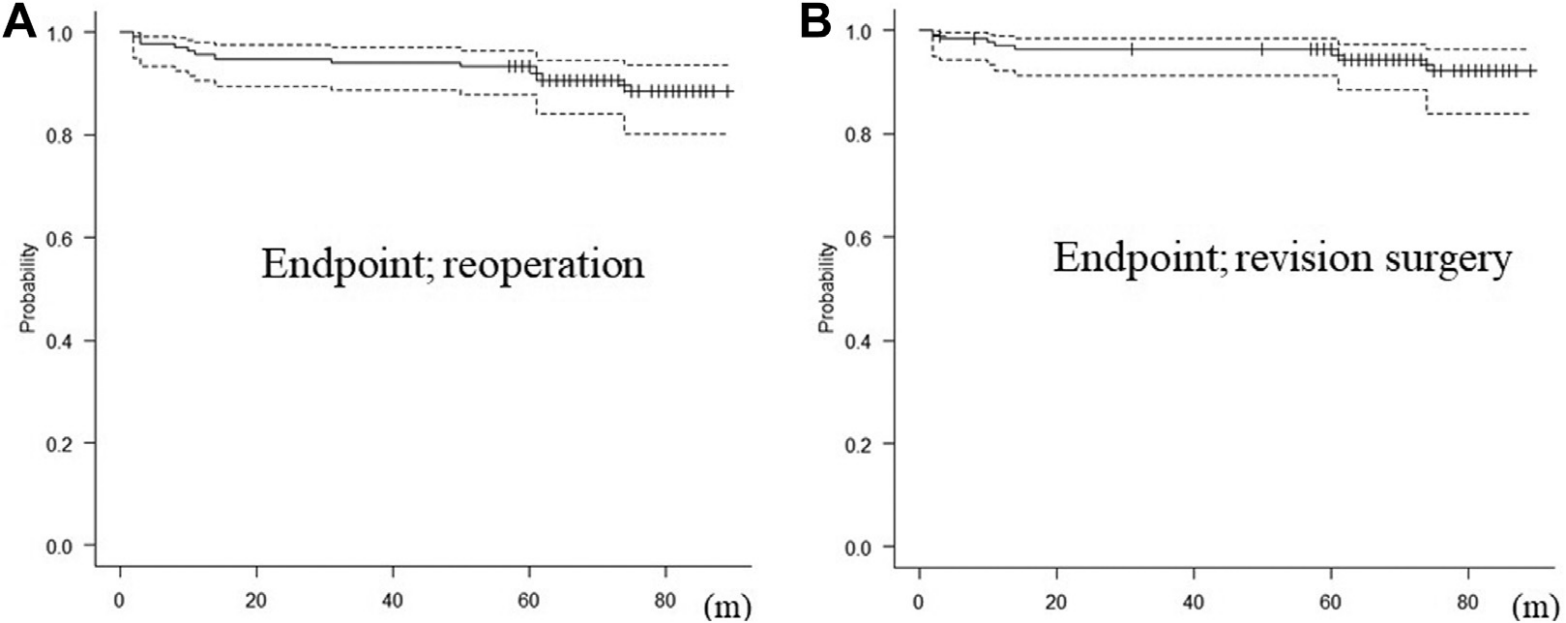
The 5-year survival rate in 137 shoulders with at least 5 years of follow-up. (**A**) The survival rate was 93.4% (95% CI, 87.8-96.5) for reoperation, and (**B**) 96.3% (95% CI, 91.4-98.5) for revision surgery. *CI*, confidence interval.

ROM data are provided in Table III. All ROM parameters were significantly improved postoperatively (*P* < .001). Additionally, the 5-year survival rate (n = 137) was 93.4% (95% confidence interval: 87.8%-96.5%) for reoperation and 96.3% (95% confidence interval: 91.4%-98.5%) for revision surgery (Figure 1).

**Table III:** Comparisons in the range of motion pre-and postoperatively.

	Preoperative	Postoperative	*P* value
AE (mean ± SD, degrees)	62.6 ± 40.6	126.4 ± 27.4	<.001
1st ER (mean ± SD, degrees)	20.4 ± 19.0	30.2 ± 17.0	<.001
2nd ER (mean ± SD, degrees)	37.4 ± 18.6	49.2 ± 18.6	<.001
2nd IR (mean ± SD, degrees)	0.5 ± 6.3	2.2 ± 4.5	<.001
ADD (mean vertical level)	L4	L3	<.001

*AE*, anterior elevation; *SD*, standard deviation; *ER*, external rotation; *IR*, internal rotation; *ADD*, adduction.

**Figure 2:**
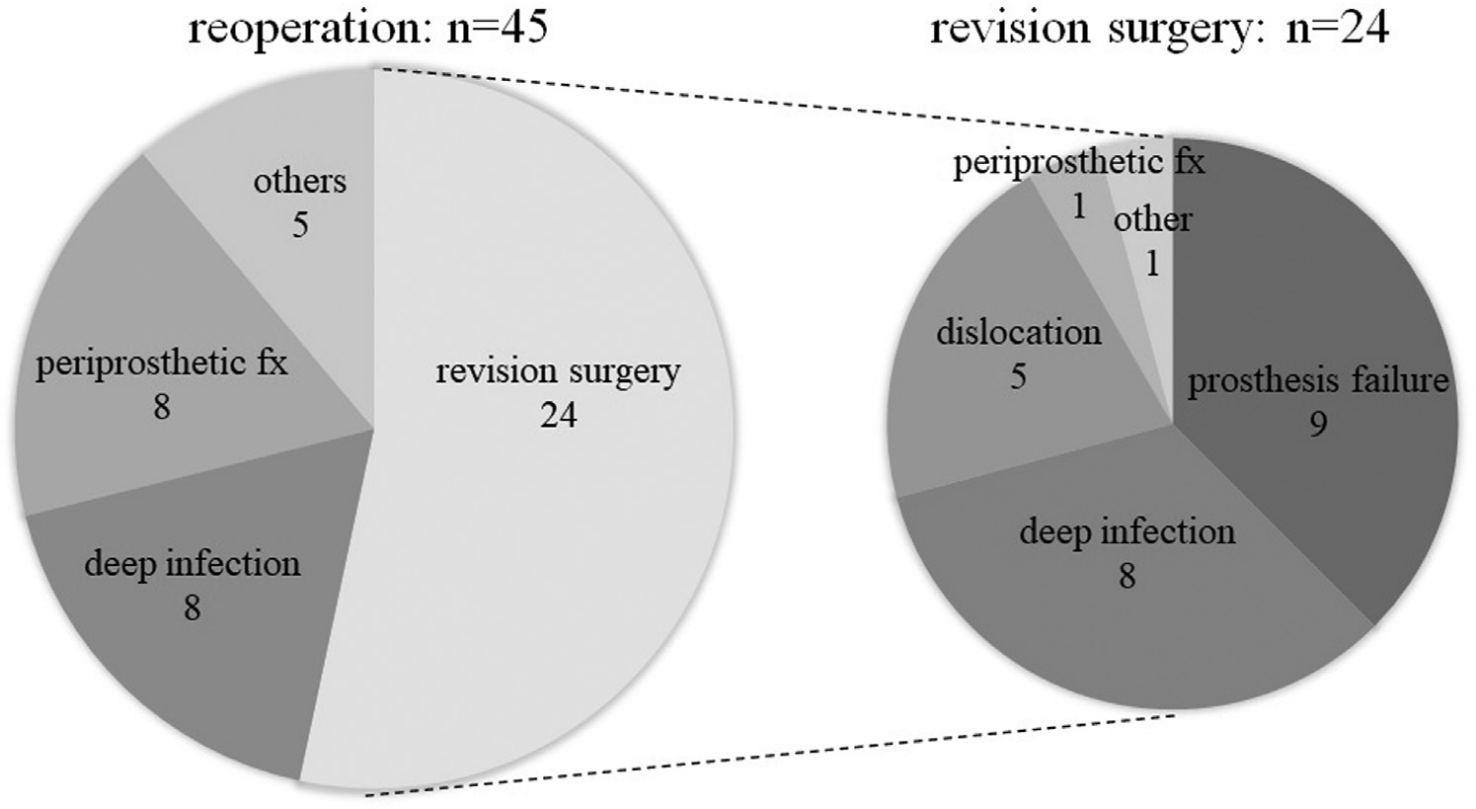
Causes of reoperation and revision surgery. All cases with prosthesis failure required revision surgery.

Complications occurred in 256 shoulders (42.0%), the details of which were shown in Table IV. Reoperation occurred in 45 shoulders (7.3%); among these, revision surgery was required in 24 shoulders (3.9%). The causes of reoperation and revision surgery are shown in Figure 2. All 9 patients with prosthesis failure (the baseplate in 8 shoulders and stem in 1) required revision surgery. Among 17 cases with neurological disorders as a complication, the affected nerve was the ulnar nerve in 8 shoulders, axillary nerve in 3, and plexus, radial, and median nerves in 2. Nine of 17 shoulders (52.9%) with neurological disorders had a persistent disorder for more than 1 year (ulnar nerve in 7 shoulders and axillary nerve in 2). On imaging, scapular notching was observed in 145 (23.6%) shoulders. According to the Sirveaux classification,^30^ the notching was grade 1 in 106 shoulders, grade 2 in 34, grade 3 in 5, and grade 4 in 0. Prosthesis loosening occurred in 13 (2.1%) shoulders, with the stem affected in 11 shoulders and the baseplate in 2. Additionally, heterotopic ossification occurred in 80 (13.0%) shoulders. Scapular notching occurred significantly more frequently with in-lay types (114 shoulders) than with on-lay types (31 shoulders) (*P* < .001). However, there were no statistically significant differences in ROM parameters between patients with and without scapular notching (Table V).

**Table IV:** Complications after reverse total shoulder arthroplasty.

Complications	No. of shoulders	Percentage of all shoulders
Scapular notching	145	23.6
Heterotopic ossification	80	13.0
Reoperation	45	7.3
Revision surgery	24	3.9
Acromial fx	24	3.9
Neurological disorder	17	2.8
Deep infection	16	2.6
Prosthesis loosening	13	2.1
Periprosthetic fx	11	1.8
Dislocation	9	1.5
Prosthesis failure	9	1.5
Clavicle fx	4	0.7
Scapular spine fx	2	0.3
Total∗	258	42.0

*fx*, fracture.

∗ With any complication.

**Table V:** Comparisons in the range of motion between patients with and without scapular notching.

	With notching (n = 145)	Without notching	*P* value
AE (mean ± SD, degrees)	122.4 ± 26.4	127.6 ± 27.6	.052
1st ER (mean ± SD, degrees)	30.8 ± 17.3	30.1 ± 16.7	.668
2nd ER (mean ± SD, degrees)	47.4 ± 17.5	49.8 ± 18.8	.261
2nd IR (mean ± SD, degrees)	2.1 ± 4.3	2.2 ± 4.6	.867
ADD (mean vertical level)	L3	L3	.310

*AE*, anterior elevation; *SD*, standard deviation; *ER*, external rotation; *IR*, internal rotation; *ADD*, adduction.

## Discussion

This retrospective multicenter study is the first large case series to investigate complications after RSA in Japan. Scapular notching was the most common complication, with a rate similar to that in previous studies.^1^^,^^33^ Additionally, the overall frequency of complications after RSA was similar to that in other countries.^4^^,^^9^^,^^10^^,^^12^^,^^19^^,^^25^^,^^27^^,^^33^ Therefore, the impact of racial differences on complications after RSA may be small. However, the rate of revision surgery (3.9%) was slightly lower than that in previous studies (4.2%∼10.1%).^3^^,^^4^^,^^25^^,^^33^ This difference might be due to the shorter follow-up duration in the present study and restricted guidelines and certifications in Japan.

Scapular notching occurred significantly more frequently with in-lay types than with on-lay types, consistent with previous studies.^15^^,^^16^ Sirveaux classification^30^ grade 1 or 2 cases comprised the majority (96.6%), and there were no grade 4 cases. However, scapular notching has been reported to worsen gradually over time^8^; since the average follow-up period in the present study was relatively short (approximately 4 years), further follow-up is necessary. The influence of scapular notching on clinical outcomes has not been fully clarified, with some studies showing no difference in clinical outcomes between patients with and without notching and others showing worse anterior elevation angles in those with notching.^6^^,^^29^ In the present study, there was no statistically significant difference in ROM parameters between patients with and without scapular notching. However, additional studies that include clinical scores are necessary.

Acromial fractures are relatively common (3.9% in the present study) and are known as a unique complication that occurs due to deltoid overload with lengthening after RSA.^9^^,^^22^ Levy et al reported that CT is better than radiography for diagnosing acromial fractures.^18^ Therefore, it is possible that the frequency would be even higher if CT examinations were routinely performed. Furthermore, acromial fractures are considered to be associated with poor clinical outcomes^9^^,^^22^^,^^23^; thus, further studies are necessary to examine the risk factors and evaluate clinical outcomes, including clinical scores. Neurological disorders occurred in 17 shoulders (2.8%), approximately half of which persisted for more than 1 year. The rate of postoperative neurological disorders in RSA is reported to range from 0.5% to 4%, and neurological disorders are associated with poor clinical outcomes.^7^^,^^28^^,^^33^ Therefore, intraoperative neuromonitoring has been used to reduce the incidence of neurological disorders after RSA^28^ and further studies are necessary to prevent neurological disorders.

All ROM parameters were significantly improved after RSA, including ER and IR, and the 5-year survival rate was 93.4% for reoperation and 96.3% for revision surgery; thus, the clinical outcomes of RSA were good at an average of 4 years after RSA. However, several studies have reported that clinical outcomes after RSA worsen over time owing to deltoid fatigue.^3^^,^^24^ Bacle et al reported that relative and absolute Constant Scores at the long-term follow-up evaluation (mean, 150 months) decreased significantly compared to scores at the medium-term follow-up evaluation (mean, 40 months).^3^ In contrast, Schoch et al reported that progressive deterioration of overhead ROM occurs at a slightly greater rate than that observed in natural shoulders^26^; however, the difference was not severe. Based on these studies, a longer follow-up of more than 10 years is necessary to accurately evaluate the clinical outcomes of RSA. Although the main causes of reoperation in the present study were deep infection and periprosthetic fracture, all cases with prosthesis failure, 5 of 9 (55.6%) cases with dislocation, and 8 of 16 (50%) cases with deep infection required revision surgery. Therefore, attention should be paid to preventing these complications and achieving good clinical outcomes.

This study has several limitations. First, we could not evaluate clinical scores because different scores were used at each hospital in this multicenter study. Second, the surgical indications and procedures were not consistent. Third, this was a retrospective study, without randomization. Fourth, the rehabilitation protocol differed among hospitals; however, all patients used an abduction sling for 2-3 weeks postoperatively. Fifth, this study included data from the start of the adoption of RSA in Japan; thus, an effect of the learning curve is unequivocal.

## Conclusion

We evaluated the complications after RSA in Japan. The frequency of complications after RSA in this study was similar to that in other countries; thus, the impact of racial differences might be small. Furthermore, the clinical outcomes of RSA were good at an average of 4 years after RSA.

## Acknowledgments

The authors would like to thank Editage (www.editage.com) for English language editing.

## Disclaimers

Funding: No funding was disclosed by the authors.

Conflicts of interest: The author, their immediate family, and any research foundation with which they are affiliated have not received any financial payments or other benefits from any commercial entity related to the subject of this article.
